# Relationship between cytokine release and stress hyperglycemia in patients hospitalized with COVID-19 infection

**DOI:** 10.3389/fmed.2022.988686

**Published:** 2022-08-19

**Authors:** Andrea Da Porto, Carlo Tascini, Gianluca Colussi, Maddalena Peghin, Elena Graziano, Chiara De Carlo, Luca Bulfone, Martina Antonello, Emanuela Sozio, Martina Fabris, Francesco Curcio, Carlo Pucillo, Cristiana Catena, Leonardo A. Sechi

**Affiliations:** ^1^Division of Internal Medicine, Department of Medicine, University of Udine, Udine, Italy; ^2^Division of Infectious Diseases, Department of Medicine, University of Udine, Azienda Sanitaria Universitaria Friuli Centrale (ASUFC), Udine, Italy; ^3^Division of Laboratory Medicine, University of Udine, Azienda Sanitaria Universitaria Friuli Centrale (ASUFC), Udine, Italy; ^4^Laboratory of Immunology, Department of Medicine, University of Udine, Udine, Italy

**Keywords:** cytokines, COVID-19, new onset diabetes, Stress Hyperglycemia Ratio, outcomes, humoral immune response, immunoparalysis

## Abstract

**Introduction:**

Stress hyperglycemia is a frequent finding in patients with COVID-19 infection and could affect the outcome of disease. Cytokines released in response to infection could have adverse effects on insulin sensitivity and pancreatic beta-cell function. The aim of the study was to examine the relationships of stress hyperglycemia with cytokines and clinical outcomes in hospitalized patients with COVID-19.

**Methods:**

In a cross-sectional analysis of 150 patients hospitalized for COVID-19 infection who were included in the GIRA-COVID database, we identified patients with stress hyperglycemia by calculation of the Stress Hyperglycemia Ratio (SHR) and use of a cut-off of 1.14. Plasma levels of cytokines principally involved in COVID-19 infection-related cytokine storm were measured. Outcome variables were use of mechanical ventilation and death within 60 days from hospital admission.

**Results:**

Patients with SHR > 1.14 had significantly higher plasma insulin, HOMA-index, and levels of interleukin-10 (IL-10), interleukin-10/tumor necrosis factor-a ratio (IL-10/TNF-α), and CXC motif chemokine ligand 10 (CXCL10) than patients with SHR ≤ 1.14. IL-10, IL-10/TNF-α ratio, CXCL10, and IFN-γ were significantly and directly related with SHR in univariate analysis and multivariate logistic regression models showed that IL-10, IL-10/TNF-α ratio, and CXCL10 were independently associated with SHR>1.14. In a multivariate logistic model, stress hyperglycemia predicted use of mechanical ventilation (OR 2.453; CI 1.078–6.012) and death (OR 2.281; CI 1.049–7.369) independently of diabetes and other major confounders.

**Conclusions:**

In patients hospitalized for COVID-19 infection, stress hyperglycemia is associated with worse clinical outcomes and is independently related to levels of cytokines that might impair glucose homeostasis.

## Introduction

The association between acute hyperglycemia and worse outcomes in acute care settings is well known (1, 2). Studies conducted in non-diabetic subjects admitted to medical wards (3) and intensive care units (4) have shown that stress hyperglycemia is an independent predictor of prolonged hospital stay and in-hospital mortality. It is known also that critically ill patients frequently develop hyperglycemia upon hospital admission regardless of pre-existing diabetes. Recent evidence demonstrates elevated prevalence of stress hyperglycemia and new onset diabetes in patients hospitalized with SARS-CoV-2 infection. This prevalence appears disproportionately high when it is compared with what seen with other acute illnesses (5). Previous studies suggest that hyperglycemia on admission is a strong predictor of poor outcome among patients hospitalized with SARS-CoV-2 infection (6). On the other hand, in patients with type 2 diabetes, SARS-CoV-2 infection has been associated with rapid worsening of glycemic control possibly leading to ketoacidosis or hyperosmolar hyperglycemia (7, 8).

To date, no clear answer exists to explain why SARS-CoV-2 infection causes stress hyperglycemia with such high frequency. Although almost any acute inflammatory state impairs insulin sensitivity and glucose disposal through activation of counter-regulatory hormones and proinflammatory cytokines (9, 10), this effect appears to be more pronounced in patients with SARS-CoV-2 infection. This might hypothetically be related to the unrestrainable cytokine storm that occurs in patients with severe SARS-CoV-2 infection (11). Many cytokines and chemokines that are released during the acute phase response to SARS-CoV-2 infection (12) could possibly damage pancreatic beta-cells and impair insulin sensitivity, but their relationship with stress hyperglycemia is still unknown. The aim of this study was to examine the relationships between stress hyperglycemia and circulating cytokines and their relevance for clinical outcomes in patients hospitalized with SARS-CoV-2 infection.

## Methods

This is a cross-sectional analysis of the GIRA-COVID database that was a prospective, observational, study conducted in 2021 at the University Hospital-University of Udine, a quaternary acute care regional hospital serving an area of more than 700,000 people in northeast of Italy. We examined clinical, laboratory and radiological characteristics, and 60-day outcomes of 200 consecutive COVID-19 patients who were hospitalized from January 1st to February 28th, 2021. All patients had presented with fever and/or respiratory symptoms at specific COVID-19 points of care that were set up in the Infectious Disease and the Emergency Units of the Hospital. Diagnosis of COVID-19 infection was established for confirmed (patients with pneumonia and a positive nucleic acid amplification test for SARS-CoV-2 in respiratory tract specimens) and suspected (patients with pneumonia with typical laboratory findings and/or lung CT scan imaging and/or positive serology but negative SARS-CoV-2 nasopharyngeal swab) cases.

Patients' data were collected from the University hospital electronic database. The database is continuously and timely updated and contains all the data of patients registered to the hospital, including patients' demographic, anthropometric, and clinical variables, laboratory test results, historical and current diagnoses, together with history of previous hospitalizations, and historical and current medication list. Clinical severity of COVID-19 disease was assessed at admission according to the World Health Organization (WHO) classification. CT scan of the lung was performed on hospital admission in patients with severe symptoms and within 72 h from admission in patients with mild symptoms. The study was conducted according to the guidelines of the Declaration of Helsinki and approved by the Local Ethics Committee (protocol 14180/O/GEN/ARCS on 19/04/2021). All patients gave their written informed consent to the study.

### Laboratory data

Identification of SARS-CoV-2 infection was based on the detection of unique sequences of virus RNA by nucleic acid amplification tests (NAAT) such as real-time PCR (RT-PCR) on a nasopharyngeal swab. The SARS-CoV-2 E gene was used for screening and RdRp and N genes for confirmation. The viral RNA was extracted by using automated RNA extraction with the ELITe InGenius® SP200 System (ELITechGroup 92800 Puteaux • France) and RT-qPCR was performed using a LightMix® Modular SARS and Wuhan CoV E-gene kit on a LightCycler® 480 II instrument (Roche). The specimens were considered positive if the cycle threshold value for at least one of the three genes was ≤ 36. The RT-PCR was conducted as recommended by the WHO for COVID-19 clinical management and outbreak control purposes.

At hospital admission and before starting intensive insulin treatment, venous blood samples were obtained for measurement of blood glucose, glycated hemoglobin (HbA1c), plasma insulin and lipids, C-reactive protein (CRP), and pro-adrenomedullin. A magnetic bead-based multiplex assay (Bio-Plex Pro™ Custom Human Cytokines and Chemokine Panel, procarta-Plex, Bio-Rad Laboratories, San Jose, California, 95134 USA) quantified several cytokines and chemokines: interleukin-1β (IL-1β, normal range <0.16 pg/ml), interleukin-6 (IL-6, normal range <7 pg/ml), interleukin-8 (IL-8, normal range 7–16 pg/ml), tumor necrosis factor-α (TNF-α, normal range 8–12 pg/ml), C-X-C motif chemokine ligand 10 (CXCL10, normal range 37–222 pg/ml), interferon-γ (IFN-γ, normal range <0.99 pg/ml), interleukin-10 (IL-10, normal range 1.8–3.8 pg/ml), interleukin-2α (IL-2α, normal range 440–1,435 pg/ml). All the other laboratory biomarkers were evaluated using routine certified diagnostic methods.

### Definition of stress hyperglycemia and insulin resistance

The Stress Hyperglycemia Ratio (SHR) was used for assessment of stress hyperglycemia. SHR is the ratio between blood glucose on admission and the previous average glucose level that is estimated from glycated hemoglobin (HbA1c) value using the equation 1.59 × HbA1c – 2.59, as proposed by Roberts et al. (13). Stress hyperglycemia was considered to be present when SHR was >1.14 according to the previous literature (13). The Homeostatic Model Assessment index (HOMA-IR) was used as an estimate of insulin sensitivity [fasting serum insulin (μU/ml) × fasting plasma glucose (mmol l-1)/22.5]. The triglyceride/glucose (TyG) index was also defined as log [fasting triglycerides (mg/dl) × fasting glucose (mg/dl)/2].

### Statistical analysis

Values of normally distributed variables are expressed as mean ± standard deviation and skewed variables as median and interquartile range. Normality of distribution of the study variables was assessed by the Kolmogorov–Smirnov test and skewed variables were analyzed after logarithmic conversion. Pearson's chi-squared test was used to compare frequency distributions. Spearman's correlation coefficient was used to express the correlation between cytokines and stress hyperglycemia. Logistic regression analysis was performed to identify which variables were independent predictors of a SHR of more than 1.14. In this analysis, variables were sequentially entered in a stepwise model according to the strength of statistical significance obtained in the univariate analysis. The association between stress hyperglycemia and clinical outcomes was evaluated in a multivariate logistic regression model where stress hyperglycemia was included either as values of SHR >1.14 or SHR tertiles. A two-tailed probability value of less than 5% was considered to indicate statistical significance. Data analyses were performed using XLSTAT 2020 (Addinsoft, New York, NY, USA).

## Results

Over the study period, 200 patients were admitted to our clinic with a confirmed diagnosis of COVID-19 infection. Overall, 150 patients had complete data for assessment of SHR and cytokines and chemokines measurements. The clinical characteristics of the study patients are shown in Table 1 where patients are grouped according to presence or absence of stress hyperglycemia. Age of patients included in the study was 69.9 ± 12.4 years, 35 % were male, and body mass index (BMI) and waist circumference were 28.2 ± 5.4 kg/m^2^ and 103 ± 13 cm, respectively. Hypertension was present in 59% of patients, 37% had type 2 diabetes mellitus, and 31% had history of cardiovascular disease. Blood glucose on admission was 6.5 mmol/l (IQR 5.3–8.4) and median HbA1c 6.3% (IQR 5.8–6.8). Most patients (62%) had severe COVID-19 disease as defined by WHO stages 3–4. On admission, 4 patients (3%) were taking steroids and 4 (3%) were on insulin treatment.

**Table 1:** Clinical characteristics and biochemical variables in patients hospitalized with COVID-19 infection who were grouped according to a Stress Hyperglicemia Ratio (SHR) ≤ 1.14 or >1.14.

**Variable**	**All patients (150)**	**SHR ≤ 1.14 (109)**	**SHR > 1.14 (41)**	***P*-value**
Age (years)	69.9 ± 12.4	69.9 ± 12.8	69.9 ± 13.3	0.825
Male sex *n* (%)	53 (35.1)	39 (35.7)	14 (34.1)	0.881
BMI (kg/m^2^)	28.3 ± 5.4	28.4 ± 5.8	28.1 ± 5.6	0.643
Waist Circ. (cm)	103.4 ± 13.3	103.6 ± 13.4	102.6 + 15.3	0.720
WHO 1–2 *n* (%)	57 (38)	41 (69)	16 (25)	0.332
WHO 3–4 *n* (%)	93 (62)	33 (67)	27 (54)	0.268
Hypertension *n* (%)	88 (58.6)	63 (55.1)	23 (60)	0.897
Diabetes *n* (%)	56 (37.3)	42 (37.2)	16 (42)	0.925
CV disease *n* (%)	47 (31.3)	31 (29)	16 (39)	0.213
Liver disease *n* (%)	13 (8.6)	7 (6.1)	3 (6)	0.495
Malignancy *n* (%)	18 (12)	13 (12)	5 (12)	0.880
Background steroids *n* (%)	4 (2.6)	3 (2)	1 (4)	0.767
Statins *n* (%)	35 (23.3)	21 (20)	14 (34)	0.005
Background insulin *n* (%)	4 (2.6)	3 (2.5)	1 (2.5)	0.643
Creatinine (mg/dl)	1.2 ± 1.2	1.1 ± 0.7	1.4 ± 2	0.141
Urea (mg/dl)	24.9 ± 12.9	24.9 ± 13.7	24.8 ± 10	0.509
AST (UI/l)	42.5 ± 27.6	40.7 ± 31	44.3 ± 32	0.203
ALT (UI/l)	42.2 ± 31.6	40.1 ± 33.9	43.1 ± 27.7	0.541
LDH (UI/l)	693.7 ± 302	659 ± 260	783 ± 379	0.049
CPK (UI/l)	167 (35–169)	154 (44–167)	202 (33–166)	0.417
Uric acid (mg/dl)	4.8 ± 1.8	4.7 ± 2	5.3 ± 1.4	0.384
Cholesterol (mg/dl)	150.1 ± 12	154 ± 39.5	136.2 ± 36.7	0.020
HDL-Cholesterol (mg/dl)	33.4 ± 9.2	34.1 ± 9.6	32.7 ± 9.7	0.792
LDL-Cholesterol (mg/dl)	87.7 ± 33.5	89 ± 33.1	74.2 ± 31.9	0.036
Triglycerides (mg/dl)	162.1 + 58.8	160.4 ± 61.8	156.9 ± 58.9	0.129
Glucose (mmol/l)	6.5 (5.2–6.4)	5.8 (4.8–6.9)	10.3 (7.1–12.7)	<0.001
HbA1c (DCCT %)	6.5 ± 1.1	6.5 ± 1.2	6.4 ± 1.1	0.328
HbA1C (IFCC mmol/mol)	48 ± 11	48 ± 10	46 ± 11	
Insulin (nUI/ml)	20.9 ± 19.6	17.6 ± 14	29.9 ± 28	0.008
HOMA-IR	6.8 ± 7.6	4.7 ± 3.9	13.1 ± 12.1	<0.001
TyG Index	4.9 ± 0.4	4.8 ± 0.26	5.1 ± 0.29	<0.001
SHR	0.94 ± 0.35	0.8 ± 0.1	1.5 ± 0.39	<0.001

*WHO, World Health Organization; CV, cardiovascular; AST, aspartate aminotransferase; ALT, alanine aminotransferase; LDH, lactate dehydrogenase; CPK, creatine phosphokinase; LDL, low-density lipoprotein; HDL, high-density lipoprotein; HbA1c, glycated hemoglobin; HOMA-IR, homeostatic model assessment index; TyG, triglyceride/glucose index; SHR, stress hyperglycemia ratio*.

Forty-one (27%) of 150 patients had stress hyperglycemia as defined by a SHR of more than 1.14. Patients with stress hyperglycemia had significantly higher plasma insulin, HOMA-index, TyG index, and lactate dehydrogenase and lower total and LDL-cholesterol levels than patients without stress hyperglycemia. No significant differences in demographic and anthropometric characteristics, frequency of comorbidities, COVID-19 severity on hospital admission, renal function, and liver tests were observed between patients with or without stress hyperglycemia.

Table 2 reports the levels of cytokines and chemokines that were measured in the study patients. Patients with stress hyperglycemia had significantly higher levels of IL-10, IL-10/TNF-α ratio, CXCL10, and CRP than patients without stress hyperglycemia.

**Table 2:** Plasma levels of cytokines and chemokines in patients hospitalized with COVID-19 infection who were grouped according to a Stress Hyperglicemia Ratio (SHR) ≤ 1.14 or >1.14.

**Variable**	**All patients (150)**	**SHR ≤ 1.14 (109)**	**SHR >1.14 (41)**	***P*-value**
IL-1β (pg/mL)	0.002 (0–0.078)	0.001 (0–0.097)	0.001 (0–0.014)	0.245
sIL-2R-α (pg/mL)	3,899 (2,857–5,449)	3,895 (2,870–5,197)	4,002 (2,770–5,653)	0.546
IL-6 (pg/mL)	31.9 (18.5–69.1)	35.4 (18.5–84.5)	29.5 (20–52)	0.333
IL-8 (pg/mL)	35.6 (24.9–52.6)	34.6 (25–52)	35.3 (20–55)	0.433
IL-10 (pg/mL)	15.1 (9–23.1)	13.1 (7–19)	19.8 (15–27)	<0.001
IL-10/TNFα ratio	0.9 (0.5–1.3)	0.8 (0.5–1.2)	1.2 (0.8–1.6)	<0.001
CXCL10 (pg/mL)	1,193 (738–1,778)	1,011 (563–1,626)	1,515 (1,155–1,972)	<0.001
TNF-α (pg/mL)	16.4 (13–21.4)	16.1 (13–20)	17.9 (13–24)	0.819
IFN-γ (pg/mL)	1.6 (0.4–3.9)	1.3 (0.3–3.4)	1.8 (1–4.8)	0.063
CRP (mg/dl)	89 (36–114)	63 (30–100)	104 (61–168)	<0.001
Pro-ADM (pg/mL)	1.3 ± 0.8	1.4 ± 1.2	1.15 ± 0.5	0.111
Pro-CT (ug/mL)	0.11 (0.06–0.2)	0.1 (0.06–0.2)	0.15 (0.1–0.3)	0.502
D-Dimer	753 (488–1,368)	740 (469–1,361)	793 (513–1,497)	0.478

*IL-1β, interleukin-1β; IL-6, interleukin-6; IL-8, interleukin-8; TNF-α, tumor necrosis factor-α; CXCL10, C-X-C motif chemokine ligand 10; IFN-γ, interferon-γ; IL-10, interleukin-10; sIL-2R-α, Soluble receptor of interleukin-2α; CRP, C-reactive protein; Pro-CT, procalcitonin; Pro-ADM, pro-adrenomedullin*.

Univariate regression analysis of the relationships of plasma cytokines and chemokines with SHR is shown in Table 3. Analysis showed that IL-10, IL-10/TNF-α ratio, CXCL10, IFN-γ, and CRP were significantly and directly related with SHR values. In multivariate logistic regression models with SHR >1.14 as the dependent variable, log IL-10, IL-10/TNF-α ratio, log CXCL10, and log IFN-γ were separately included together with age, sex, presence of diabetes, CRP, and severity of disease as covariates. As shown in Table 4, log IL-10, IL-10/TNF-α ratio, and log CXCL10, but not log IFN-γ were independently associated with stress hyperglycemia.

**Table 3:** Univariate regression analysis of plasma cytokines and chemokines with Stress Hyperglycemia Ratio in patients hospitalized with COVID-19 infection.

**Variables**	**R**	***P* value**
IL-1β	−0.088	0.294
sIL-2R-α	0.159	0.057
IL-6	−0.104	0.211
IL-8	−0.004	0.958
IL-10	0.374	<0.001
IL-10/TNF-α ratio	0.363	<0.001
CXCL10	0.338	<0.001
TNF-α	0.043	0.608
IFN-γ	0.173	0.038
CRP (mg/dl)	0.320	<0.001
Pro-ADM (pg/mL)	0.119	0.166

*IL-1β, interleukin-1β; IL-6, interleukin-6; IL-8, interleukin-8; TNF-α, tumor necrosis factor-α; CXCL10, C-X-C motif chemokine ligand 10; IFN-γ, interferon-γ; IL-10, interleukin-10; sIL-2R-α/sCD25, soluble receptor of interleukin-2α; CRP, C-reactive protein; Pro-ADM, pro-adrenomedullin*.

**Table 4:** Multivariate logistic analysis with Stress Hypeglicemia Ratio >1.14 as the dependent variable.

**Variable**	**Standardized coefficient (95% confidence interval)**	**Odds ratio (95% confidence interval)**	***P* value**
Log IL-10	0.352 (0.118–0.587)	6.74 (1.9–24)	0.003
IL-10/TNF-α ratio	0.331 (0.06–0.58)	1.55 (1.08–2.11)	0.016
Log CXCL10	0.430 (0.162–0.698)	14.5 (2.5–77)	0.002
Log IFN-γ	0.070 (−0.139–0.279)	1.197 (0.71–2.1)	0.511

*IL-10, interleukin-10; TNF-α, tumor necrosis factor-α; CXCL10, C-X-C motif chemokine ligand 10; IFN-γ, interferon-γ*.

*Log IL-10, IL-10/TNF-α ratio, log CXCL10, and log IFN-γ were separately included as independent variables together with age, sex, presence of diabetes, C-reactive protein, and severity of disease*.

At the end of follow-up 32 (21.3%) patients died and 27 (18%) needed invasive mechanical ventilation in ICU. A significantly higher proportion of deaths (36.6% vs. 15.6% *p* < 0.0001) and patients needing invasive mechanical ventilation (34.1% vs. 11.9% *p* < 0.0001) was observed in patients with stress hyperglicemia. In multivariate logistic regression models, we analyzed the association of stress hyperglycemia with use of mechanical ventilation or death occurring within 60 days from hospital admission that were respectively included as the dependent variables (Table 5). Stress hyperglycemia was included in the model as an independent variable alternatively as a SHR value > 1.14 or as SHR tertiles. After correction for age, sex, BMI, presence of diabetes, and severity of COVID-19 stress hyperglycemia was associated with greater use of mechanical ventilation and higher mortality.

**Table 5:** Multivariate logistic models with use of mechanical ventilation (top panel) or death (bottom panel) occurring within 60 day from hospital admission as the dependent variables.

	**Odds ratio (95% confidence interval)**	***P* value**
**Mechanical ventilation**		
SHR > 1.14	2.453 (1.078–6.012)	<0.001
Tertile I SHR	reference	–
Tertile II SHR	2.283 (0.833–6.257)	0.109
Tertile III SHR	6.107 (2.348–15.874)	<0.001
**Death**
SHR >1.14	2.781 (1.049–7.369)	<0.001
Tertile I SHR	reference	–
Tertile II SHR	3.259 (1.183–8.979)	0.022
Tertile III SHR	5.149 (1.908–13.891)	<0.001

*Stress Hyperglycemia Ratio (SHR) >1.14 or SHR tertiles were separately included as independent variables together with age, sex, presence of diabetes, C-reactive protein, and severity of disease*.

## Discussion

The findings of this study indicate that stress hyperglycemia occurs in more than a quarter of patients who are hospitalized with COVID-19 infection. We observed higher proportion of patients taking Statins in those with Stress Hyperglicemia. We belive that this difference is due to a slight higher prevalence of patients with Hypertension and CV disease in Stress Hyperglicemia Group (Table 1). Patients with stress hyperglycemia have higher plasma insulin levels and evidence of reduced insulin sensitivity, as suggested by higher HOMA and TyG index, that is independent of preexisting diabetes. In these patients, severity of infection on hospital admission does not differ from patients without stress hyperglycemia, whereas circulating levels of some cytokines that are potentially linked to glucose metabolism are significantly increased. Also, and most important, presence of stress hyperglycemia is an independent predictor of the clinical outcome of COVID-19 patients, both in terms of likelihood of need of mechanical ventilation and death.

Previous studies reported that stress hyperglycemia as assessed by SHR is a better prognostic indicator of clinical outcome than simple blood glucose measurement in patients with stroke (14), sepsis (2), trauma (15) acute myocardial infarction (16), and in patients admitted to general medical wards for acute illness (3). Previous studies have also reported that stress hyperglycemia is associated with poorer outcomes in patients hospitalized with COVID-19 infection (17). Differently, long-term glucose control as assessed by HbA1c levels has no association with the clinical outcome in diabetic patients hospitalized with COVID-19 (18). Our results support these observations strengthening the view that acute stress hyperglycemia has greater impact on the clinical outcome of COVID-19 patients than chronic glycemic control.

### Cytokines and stress hyperglycemia

Because of the high frequency of stress hyperglycemia observed in our COVID-19 patients, we sought to analyze the possible role of several cytokines that could contribute to acute changes of blood glucose levels. In this analysis, levels of IL-10, IL-10/TNF-α ratio, and CXCL10 were found to be independent predictors of stress hyperglycemia as defined by a SHR of more than 1.14. To our knowledge, this is the first study to report independent associations between cytokines and stress hyperglycemia in patients with COVID-19 infection. This observation might have important implications, in particular for CXCL10 that, in addition to pro-inflammatory action (19), could be directly involved in glucose homeostasis. First, it was shown that CLCX10 is released abundantly by the adipose tissue in response to an acute inflammatory response induced by endotoxemia (20), thereby leading to insulin resistance under stress conditions. This might have specific relevance in patients with COVID-19 infection in whom involvement of adipose tissue has a crucial role for the spread of inflammation (21). Second, increased CXCL10 levels are frequently detected in patients with overt type 1 and type 2 diabetes and in subjects at risk of development of diabetes just before its onset (22, 23). In this view, it has been shown that activation of CXCL10 and of its specific receptor CXCR3 could impair pancreatic beta-cell function (23). In animal models of type 1 diabetes, pancreatic insulitis was associated with CXCL10 production and inhibition of the CXCL10/CXCR3 axis prevented development of autoimmune diabetes (24). Moreover, CXCL10 was reported to induce beta-cell apoptosis in type 2 diabetes (25). Along this line, it has been recently demonstrated that extracellular vesicles that are derived from pancreatic beta-cells and are exposed to pro-inflammatory cytokines impair beta-cell activity by activation of the CXCL10/CXCR3 axis (26). Thus, the present study points to an important role of CXCL10 for induction of stress hyperglycemia in COVID-19.

In our patients, stress hyperglycemia was also found to be independently associated with higher levels of IL-10 and by its ratio to TNF-α. IL-10 is a Th2-type cytokine that is produced by a wide range of immunological cell types and has potent anti-inflammatory activity through inhibition of cytokine production (e.g., TNF-α, IL-6) and activity (27). Although the anti-inflammatory action of IL-10 was initially regarded as a beneficial event potentially limiting the damage caused by the systemic inflammatory response, some evidence suggests that an IL-10 excess could be associated with worse clinical outcomes. A role of IL-10 and IL-10/TNF-α ratio as markers of immune paralysis in prediction of adverse outcome was demonstrated in studies conducted in septic patients (28). Moreover, in agreement with our findings, it was reported that patients with stress hyperglycemia during severe sepsis had higher serum levels of IL-10 and worse clinical outcomes when compared to those with normoglycemia or pre-existing diabetes (29).

Other studies suggested that IL-10 might increase insulin sensitivity and blocks the adverse effects of inflammatory cytokines on insulin signaling and glucose homeostasis (30). Therefore, the association observed between IL-10 and stress hyperglycemia might be hypothetically explained as a counter regulatory response to insulin resistance and stress hyperglycemia induced by acute illness (31). Significant positive correlations of IL-10 and other cytokines found in this study would possibly support this hypothesis.

Major strengths of this study are the prospective design and availability of a large database containing clinical, biochemical, and imaging information that permit accurate evaluation of possible relationships of stress hyperglycemia and cytokines with COVID-19 outcomes. There are also some important limitations. First, we included only patients who were hospitalized in a general ward setting and therefore it is not possible to extrapolate findings to other settings of care, such as home or intensive care units. Second, we could evaluate insulin sensitivity only indirectly and we do not have direct markers of insulin secretion. Third we do not have data relative to SARS-CoV2 variant that hits our patients.

### Conclusions

The findings of this study demonstrate that stress hyperglycemia is associated with more frequent need of mechanical ventilation and higher mortality in hospitalized patients with COVID-19 infection independently of diabetes. Results suggest that CXCL10 could have a role in triggering the acute hyperglycemic state in these patients. Further studies will be needed to better understand the complex interactions between cytokines and metabolic disfunction in COVID-19 patients.

## Data availability statement

The raw data supporting the conclusions of this article will be made available by the authors, without undue reservation.

## Ethics statement

The studies involving human participants were reviewed and approved by protocol 14180/O/GEN/ARCS on 19/04/2021. The patients/participants provided their written informed consent to participate in this study.

## Author contributions

AD and MP: design study. MF, CP, and FC: laboratory analysis. MA, LB, EG, and CD: practical performance. GC and AD: data analysis. AD and LS: preparation of manuscript. CT, ES, and CC: critical review. All authors have read and agreed to the published version of the manuscript.

## Conflict of interest

The authors declare that the research was conducted in the absence of any commercial or financial relationships that could be construed as a potential conflict of interest.

## Publisher's note

All claims expressed in this article are solely those of the authors and do not necessarily represent those of their affiliated organizations, or those of the publisher, the editors and the reviewers. Any product that may be evaluated in this article, or claim that may be made by its manufacturer, is not guaranteed or endorsed by the publisher.
